# Structure of Benthic Communities along the Taiwan Latitudinal Gradient

**DOI:** 10.1371/journal.pone.0160601

**Published:** 2016-08-11

**Authors:** Lauriane Ribas-Deulofeu, Vianney Denis, Stéphane De Palmas, Chao-Yang Kuo, Hernyi Justin Hsieh, Chaolun Allen Chen

**Affiliations:** 1 Department of Life Science, National Taiwan Normal University, Taipei, 11677, Taiwan; 2 Biodiversity Program, Taiwan International Graduate Program, Academia Sinica and National Taiwan Normal University, Taipei, 11529, Taiwan; 3 Biodiversity Research Center, Academia Sinica, Nangang, Taipei, 11529, Taiwan; 4 Institute of Oceanography, National Taiwan University, Taipei, 106, Taiwan; 5 ARC Centre of Excellence for Coral Reef Studies, James Cook University, Townsville, QLD, 4811, Australia; 6 Penghu Marine Biological Research Centre, Makong, Penghu County, 880, Taiwan; Universita degli Studi di Genova, ITALY

## Abstract

The distribution and the structure of benthic assemblages vary with latitude. However, few studies have described benthic communities along large latitudinal gradients, and patterns of variation are not fully understood. Taiwan, lying between 21.90°N and 25.30°N, is located at the center of the Philippine-Japan arc and lies at the northern margin of coral reef development. A wide range of habitats is distributed along this latitudinal gradient, from extensive fringing coral reefs at the southern coast to non-reefal communities at the north. In this study, we examined the structure of benthic communities around Taiwan, by comparing its assemblages in four regions, analyzing the effects of the latitudinal gradient, and highlighting regional characteristics. A total of 25 sites, 125 transects, and 2,625 photographs were used to analyze the benthic communities. Scleractinian corals present an obvious gradient of increasing diversity from north to south, whereas macro-algae diversity is higher on the north-eastern coast. At the country scale, Taiwanese coral communities were dominated by turf algae (49%). At the regional scale, we observed an important heterogeneity that may be caused by local disturbances and habitat degradation that smooths out regional differences. In this context, our observations highlight the importance of managing local stressors responsible for reef degradation. Overall, this study provides an important baseline upon which future changes in benthic assemblages around Taiwan can be assessed.

## Introduction

Coral reefs host an extraordinarily high biodiversity [1], providing shelter to hundreds of thousands of species and considerable services to human society [2, 3]. Despite the existence of coral reefs over the last 500 million years, their ecological persistence into the future seems doubtful [4]. Global threats, such as rising seawater temperature and ocean acidification, have caused a worldwide decline of this ecosystem (review in [5, 6, 7]), which suffers repeated episodes of coral bleaching [8], reduction of the calcification rates of reef organisms [5, 9, 10], and the emergence of diseases [11–13]. In addition, local human-induced and natural stressors (*i*.*e*., habitat destruction, invasive species, pollution, and overfishing) have eroded the resilience of reefs [14, 15] and have precipitated the shift of this ecosystem from coral- to algae- (or alternative taxa) dominated communities [14, 16–19].

As the ability to limit the global temperature increase to less than 2°C by the end of the century already seems out of reach [20], coral reef organisms will have to demonstrate exceptional adaptation and acclimatization capacities in order to survive future environmental conditions [21]. Despite the short delay that might be provided by those capacities and allow some species to survive the changes, migration to more suitable habitats may be a more viable alternative for many reef organisms to escape stressful conditions. In this context, mesophotic habitats [22, 23] and high latitude locations [24–27] in some regions of the world could serve as refuges for diverse taxa. Therefore, connectivity along bathymetric and latitudinal gradients is critical for ensuring a significant source of propagules to these habitats [23].

Extending between 21.90°N and 25.30°N in latitude, Taiwan is located at the center of the Philippine-Japan arc and lies at the northern margin of coral reef development [28, 29]. Warm waters from the Kuroshio Current (KC) flow from the southern point of Taiwan along its east coast toward the Ryukyu Archipelago. Sandy bottoms and steep slopes flank the island’s west and east coasts, respectively [30]. Extensive fringing coral reefs are present along the south coast and eastern islets [30, 31]. In the west and north of Taiwan, the frequent occurrence of sea surface temperatures (SSTs) < 18°C during winter is a strong limitation to reef accretion [32]. The Penghu Archipelago, located in the Taiwan Strait, and the north coast are therefore characterized by the presence of non-reefal coral communities [31, 33]. A wide range of habitats distributed along this latitudinal gradient enables Taiwan to be considered among the ten most important marine hotspots of biodiversity in the world [34]. To date, 317 species of scleractinian corals [35, 36], 1,335 species of reef fishes [37], 150 algae species, 90 species of echinoderms, and 50 species of soft corals have been recorded in Taiwan [31]. Scleractinian corals present a gradient of increasing diversity from North to South [33], whereas macro-algae diversity is higher on the northeastern coast [31].

Reefs around Taiwan are at high risk, and the region is one of the five most threatened areas in Southeast Asia [38]. Since 1957, seawater temperatures around Taiwan have increased by 1.55°C, and this region is among the most rapidly warming areas of the world [39]. Extensive bleaching events occurred in 1998 and 2007 at Kenting [40, 41] causing, at some sites, an important decrease in coral coverage. Recovery in some cases has been interrupted by tropical storms that regularly affect Taiwan reefs [42], as well as a variety of local stressors such as outbreaks of invasive species (*Terpios* at Green Island [43], *Condylactis* in Kenting [18]), and human-induced disturbances usually related to the erratic development and use of the coastline (*i*.*e*., sedimentation, nutrient enrichment, mechanical damage, overfishing, and destructive fishing methods [40, 42, 44, 45]). By 2085, SSTs around Taiwan are predicted to further increase by 2.0–2.5°C [46]. The extent to which these changes will affect benthic communities in the absence of a better management of human activities remains unclear. In particular, since there have been no studies integrating coral community information from all the different regions of Taiwan, it is difficult to assess the role that Taiwan might play in a poleward migration scenario.

Therefore, this study aims to characterize the rocky bottom benthic communities around Taiwan. The structure of benthic communities in four regions around the island were compared by analyzing the effects of the latitudinal gradient and highlighting regional characteristics. The observed pattern in benthic assemblages was interpreted the light of the diversity gradient previously recorded for some taxa, and the information available on local disturbance factors. Finally, the role that Taiwanese benthic communities could potentially play in the context of poleward range expansion of tropical taxa is discussed.

## Materials and Methods

### Study location

**Fig 1:**
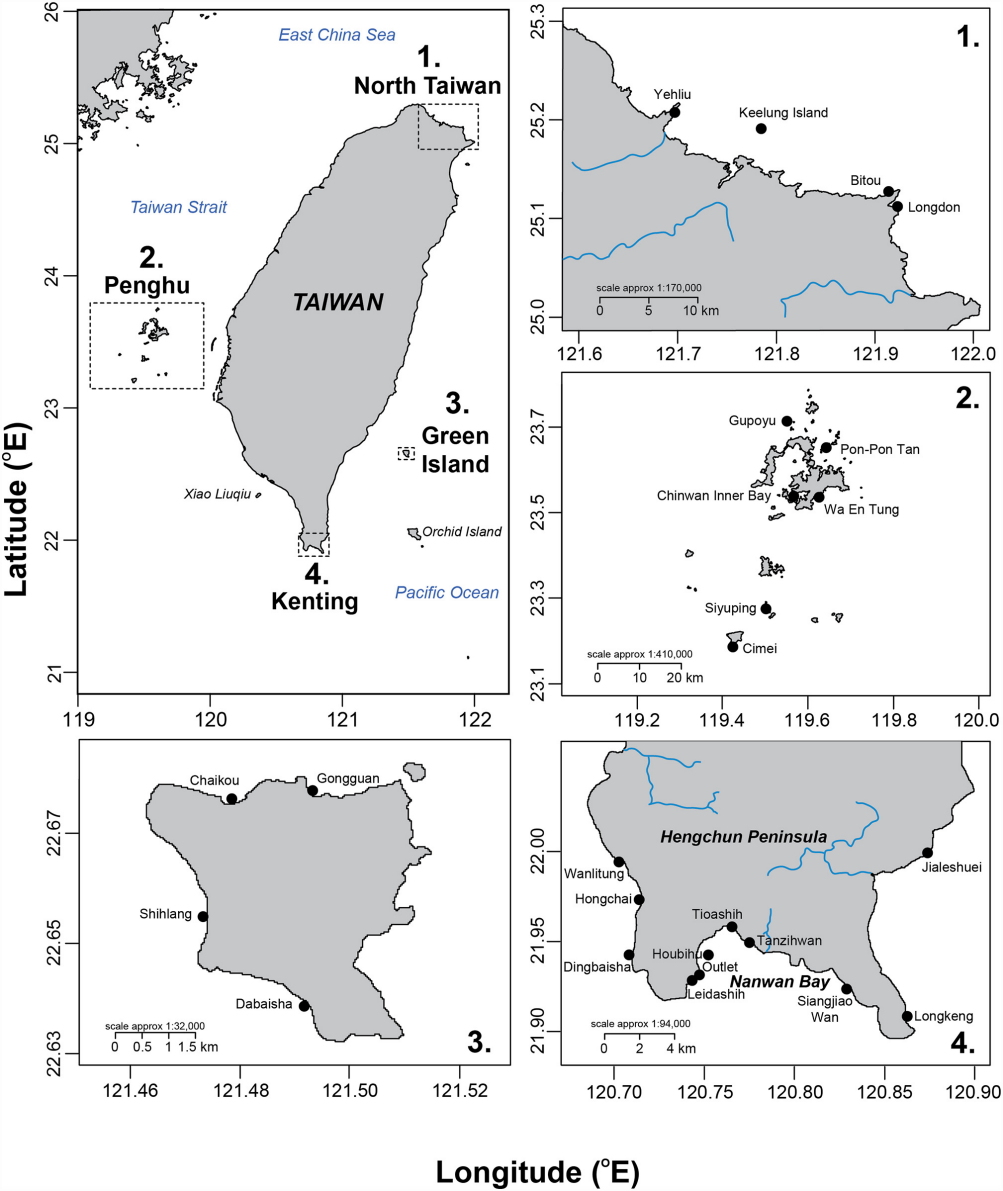
Study area. The four regions surveyed around Taiwan and locations of study sites.

Within four major regions, 25 sites around Taiwan were selected for this study, including four in north Taiwan, six at Penghu, four at Green Island, and eleven at Kenting (Fig 1). The west coast of Taiwan was not surveyed due to its sandy bottom, which is unsuitable for corals [30]. Along the east coast, the underwater topography drops steeply to a maximum depth of 4,000 m, and there are no significant coral communities here [30].

Winter seawater temperatures prevent the formation of reefs in northern Taiwan (25.17°N, 121.74°E), thus benthic coverage is limited to more-or-less well-developed coral communities [29]. Penghu (23.52°N, 119.56°E) is a small archipelago to the west of Taiwan where winter SSTs also prevent the formation of proper coral reefs, but there is a clear distinction between the tropical coral communities in the south of the archipelago and the more temperate communities in the northern islands of Penghu [47]. Northern coral assemblages suffer from high sedimentation at some locations [48], and cold shock events that decimate entire coral populations and their associated organisms [49]. Green Island (22.66°N, 121.49°E) is a small volcanic island located about 33 km off the east coast of Taiwan. With well-developed fringing reefs and clear waters, Green Island is usually considered one of the best scuba-diving destinations around Taiwan. Green Island is often impacted by typhoons, but the absence of major rivers usually restricts their impact to mechanical damage [50]. Kenting (21.90°N, 120.79°E) is located at the southern tip of Taiwan and is one of its most famous tourist spots. Its reefs attract more than five million visitors per year [40], who engage in numerous recreational activities such as scuba diving, snorkeling, surfing, and boating [44]. Kenting National Park (KNP), established in 1984 as Taiwan’s first national park, covers 33,269 hectares of terrestrial and marine environments. Some KNP sites have been particularly impacted by river run-off and mechanical damage along the path of repeated typhoons in recent decades [42]. A tide-induced upwelling [51] strongly influences physical, chemical, and biological processes in Nanwan Bay [52]. Human pressures [53], like overfishing [54] or degradation of water quality at some sites [44, 55], have negative impacts on KNP’s benthic communities.

### Sea Surface Temperatures

**Fig 2:**
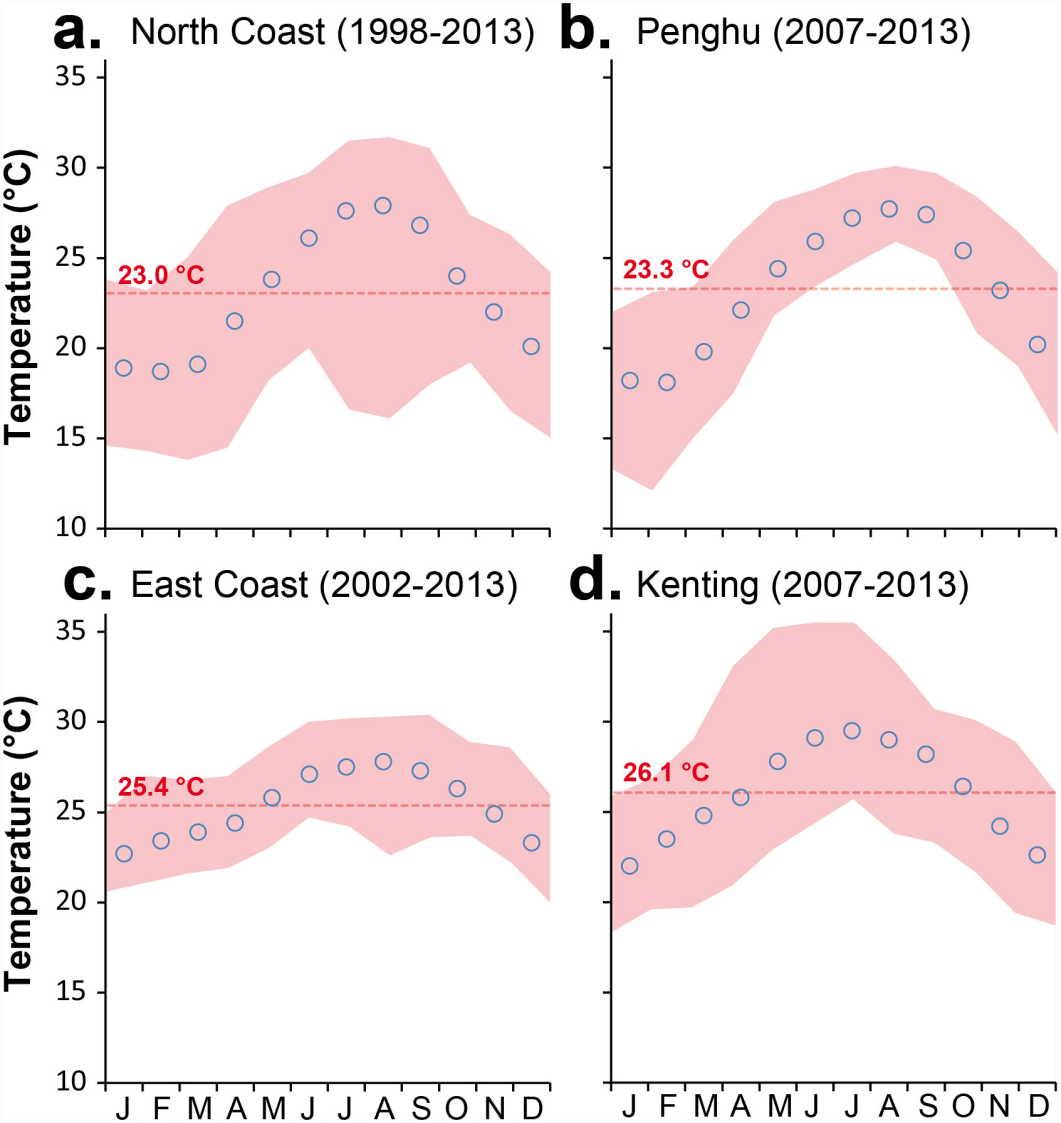
Sea Surface Temperature (SST) around Taiwan. Monthly average sea surface temperatures, annual means (red dashed line), and monthly maxima and minima (red shaded area). (a)Northern Coast (1998–2013). (b). Penghu (2007–2013). (c) Eastern coast (2002–2013). (d) Kenting (2007–2013). Data: Central Weather Bureau, Taiwan (http://www.cwb.gov.tw/).

Monthly average SSTs typically range between 22.0°C and 29.5°C at Kenting, 22.7°C and 27.8°C on the southeast coast, 18.7°C and 27.9°C on the northern coast, and 18.1°C and 27.7°C in Penghu (Central Weather Bureau of Taiwan, http://www.cwb.gov.tw/; Fig 2). Mean differences between maximum and minimum monthly temperatures (Δ°C) were 11.2 ± 2.5°C in northern Taiwan and 9.3 ± 1.8°C at Kenting, whereas the Δ°C at Green Island along the southeast coast and at Penghu did not exceed 5.8 ± 0.9°C and 7.2 ± 2.1°C, respectively. Monthly averages of less than 18°C are common in Penghu and northern Taiwan during the winter. The minimum temperature recorded in Penghu was 11.7°C in February 2008 [49]. In contrast, the maximum monthly average was recorded in June-July 2006 in Kenting, with SSTs reaching 35.5°C.

### Benthic surveys

Benthic community surveys were conducted throughout 2011–2012. Sites within each region were selected to be spatially independent of one another. The benthic assemblage was surveyed at each site using a photo-transect method. Five 20 m long line transects were randomly positioned at 5 m depth, with a minimum distance of 5 m separating each transect. Twenty-one photographs (0.5 x 0.5 m) per transect were taken at one-meter intervals, sampling a total surface of 26.25 m^2^ at each site. Benthic community structure was then assessed by analyzing the photographs. Fifty random points were positioned on each picture using CPCe v4.0 software [56], and benthic organisms were identified to the most reasonable operational taxonomic unit (OTU) possible, considering the limitation of taxonomic identification via photography (S2 File). Thirteen major categories (hard coral [HC], soft coral [SF], macro-algae [MA], encrusting coralline algae [ECA], turf algae [TU], zoanthids [ZO], sponges [SP], *Antipatharia* [AT], sea anemones [AN], ascidians [AS], other living organisms [OL], bare substrate [BS], and unknown [UN]; S5 File) were used to describe benthic coverage types. The term ‘turf algae’ used herein denotes a low-growing (several mm to cm tall) layer of mixed, filamentous algae per Connell *et al*. [57]. In addition, a category “Turf + Sediment” was defined for describing sediment deposition and characterize roughly the sedimentation at each site.

### Data analysis

Unstable substrate categories such as sand, rubble, and gravel were removed from the dataset as they do not represent suitable substrates for sessile organisms and may represent a bias when characterizing benthic communities that inhabit hard substrates. The occurrence of different OTUs along transects were summarized at country scale as well as regional, and local levels (S2 File). Data (S1 File) was converted into percentages and regional differences in major categories were tested using one-way nested analysis of variance (nested ANOVA, S3 File) from individual transects. Results of nested ANOVA are reported below Table 1. Significant *p*-values (*p* < 0.05) were interpreted using Tukey's HSD (honest significant difference) test (superscript letter in Table 1). OTU richness was calculated at the site level (*α*-diversity) and regional level (OTU richness).

**Table 1:** Composition of the benthic community around Taiwan. Mean (standard error) percent coverage of major benthic categories at the country, regional, and site levels. Results of one-way nested ANOVA* on regional differences within major categories are indicated below the table. Superscript letters indicate significant differences between regions detected by Tukey’s HSD test.

	TU*	HC*	MA*	SF*	ECA*	ZO*	SP*	AN*	AS*	AT*	OL*	UN*	BS*
**NORTH TAIWAN [104 OTUs]**	**38.9 (14.8)**^***b***^	**18.8 (9.7)**^***b***^	**23.2 (11.4)**^***a***^	**0.3 (0.4)**^***c***^	**8.3 (7.6)**^***a***^	**6.1 (11.2)**^***a***^	**0.4 (0.8)**^***ns***^	**0.2 (0.9)**^***b***^	**0.0 (0.0)**^***ns***^	**0.0 (0.0)**^***ns***^	**0.1 (0.2)**^***b***^	**0.0 (0.0)**^***ns***^	**3.7 (4.9)**^***a***^
Keelung Island	53.5 (5.1)	24.1 (7.0)	12.9 (1.1)	0.0 (0.0)	4.9 (2.3)	1.0 (1.2)	0.4 (0.3)	0.0 (0.0)	0.0 (0.0)	0.0 (0.0)	0.0 (0.0)	0.0 (0.0)	3.1 (1.0)
Yeliu	32.1 (3.8)	22.3 (11.9)	15.2 (2.9)	0.4 (0.6)	7.1 (3.7)	19.8 (16.7)	1.1 (1.5)	0.9 (1.9)	0.0 (0.0)	0.0 (0.0)	0.2 (0.1)	0.0 (0.0)	0.9 (0.6)
Bitou	44.1 (5.3)	19.0 (7.7)	28.2 (9.1)	0.4 (0.4)	4.3 (1.3)	2.5 (2.2)	0.0 (0.0)	0.0 (0.0)	0.0 (0.0)	0.0 (0.0)	0.3 (0.2)	0.0 (0.0)	1.3 (0.7)
Longdon	25.9 (20.1)	9.8 (6.5)	36.6 (7.3)	0.2 (0.5)	16.7 (11.5)	1.2 (1.5)	0.0 (0.0)	0.0 (0.1)	0.0 (0.0)	0.0 (0.0)	0.0 (0.1)	0.0 (0.0)	9.5 (7.2)
**PENGHU [132 OTUs]**	**69.8 (16.6)**^***a***^	**21.0 (18.4)**^***b***^	**2.4 (2.7)**^***c***^	**1.1 (2.1)**^***c***^	**0.8 (1.1)**^***c***^	**0.4 (1.4)**^***b***^	**0.7 (1.2)**^***ns***^	**0.0 (0.0)**^***b***^	**0.0 (0.0)**^***ns***^	**0.0 (0.0)**^***ns***^	**0.2 (0.3)**^***b***^	**0.0 (0.0)**^***ns***^	**3.5 (7.2)**^***a*,*b***^
Wa En Tung	43.5 (4.6)	54.0 (4.5)	0.6 (0.6)	0.0 (0.0)	0.7 (0.4)	0.0 (0.0)	0.9 (0.5)	0.0 (0.0)	0.0 (0.0)	0.0 (0.0)	0.1 (0.2)	0.0 (0.0)	0.1 (0.1)
Gupoyu	61.8 (12.8)	31.0 (12.8)	4.2 (2.4)	0.6 (1.0)	2.0 (1.2)	0.0 (0.0)	0.2 (0.3)	0.0 (0.0)	0.0 (0.0)	0.0 (0.0)	0.1 (0.2)	0.0 (0.0)	0.2 (0.2)
Siyuping	74.8 (7.5)	13.5 (5.5)	3.7 (3.2)	2.8 (3.5)	1.7 (1.8)	2.2 (3.0)	0.0 (0.0)	0.0 (0.0)	0.0 (0.0)	0.0 (0.0)	0.6 (0.3)	0.0 (0.0)	0.7 (1.5)
Pon Pon Tan	81.9 (5.4)	11.2 (6.2)	5.0 (3.3)	1.0 (0.7)	0.2 (0.2)	0.0 (0.0)	0.1 (0.1)	0.0 (0.0)	0.0 (0.0)	0.0 (0.0)	0.1 (0.1)	0.0 (0.0)	0.6 (0.9)
Cimei	81.0 (13.9)	10.8 (8.8)	1.1 (0.7)	2.3 (2.8)	0.2 (0.1)	0.1 (0.2)	0.1 (0.3)	0.0 (0.0)	0.0 (0.0)	0.0 (0.0)	0.1 (0.2)	0.0 (0.0)	4.4 (8.7)
Chinwan Inner Bay	76.0 (12.6)	5.4 (3.5)	0.1 (0.1)	0.0 (0.1)	0.3 (0.3)	0.0 (0.0)	3.1 (1.2)	0.0 (0.0)	0.0 (0.0)	0.0 (0.0)	0.3 (0.5)	0.0 (0.0)	14.9 (9.4)
**GREEN ISLAND [164 OTUs]**	**20.8 (14.1)**^***c***^	**39.6 (11.0)**^***a***^	**6.7 (5.4)**^***b***^	**23.8 (23.6)**^***a***^	**3.7 (2.6)**^***b***^	**0.1 (0.2)**^***b***^	**0.7 (1.1)**^***ns***^	**1.7 (3.2)**^***a***^	**0.0 (0.1)**^***ns***^	**0.0 (0.0)**^***ns***^	**0.0 (0.1)**^***b***^	**0.1 (0.2)**^***ns***^	**2.7 (3.8)**^***a*,*b***^
Gongguan	37.1 (3.7)	46.0 (4.8)	7.9 (2.0)	3.5 (2.0)	3.8 (1.0)	0.2 (0.1)	0.6 (0.9)	0.0 (0.1)	0.0 (0.0)	0.0 (0.0)	0.1 (0.0)	0.0 (0.0)	0.8 (0.6)
Chaikou	29.6 (1.3)	43.4 (7.6)	14.2 (3.6)	2.2 (1.5)	7.2 (2.1)	0.3 (0.1)	1.0 (1.4)	0.0 (0.0)	0.0 (0.0)	0.0 (0.0)	0.0 (0.1)	0.0 (0.0)	2.2 (1.5)
Dabaisha	11.3 (9.3)	42.1 (13.8)	2.7 (1.7)	33.0 (9.6)	1.5 (0.7)	0.0 (0.1)	0.2 (0.2)	2.9 (5.0)	0.1 (0.1)	0.0 (0.0)	0.0 (0.1)	0.0 (0.0)	6.2 (6.5)
Shihland	5.4 (2.1)	26.7 (4.4)	2.2 (1.2)	56.4 (4.4)	2.4 (1.6)	0.0 (0.1)	1.0 (1.6)	4.0 (2.6)	0.0 (0.0)	0.0 (0.0)	0.0 (0.0)	0.2 (0.4)	1.7 (1.9)
**KENTING [200 OTUs]**	**51.5 (26.0)**^***b***^	**24.1 (19.0)**^***b***^	**10.6 (7.7)**^***b***^	**7.8 (13.4)**^***b***^	**3.8 (3.6)**^***b***^	**0.2 (0.4)**^***b***^	**0.3 (0.7)**^***ns***^	**0.1 (0.6)**^***b***^	**0.0 (0.1)**^***ns***^	**0.0 (0.0)**^***ns***^	**0.5 (0.5)**^***a***^	**0.0 (0.0)**^***ns***^	**1.1 (2.8)**^***b***^
Houbihu	20.9 (4.5)	53.1 (13.1)	3.1 (2.7)	17.1 (11.5)	4.1 (1.3)	0.2 (0.1)	0.2 (0.2)	0.0 (0.0)	0.0 (0.0)	0.0 (0.0)	0.2 (0.3)	0.0 (0.1)	1.1 (1.2)
Outlet	27.7 (6.1)	47.8 (6.9)	8.4 (4.4)	12.8 (10.7)	1.6 (0.9)	0.0 (0.1)	0.0 (0.0)	0.0 (0.0)	0.0 (0.0)	0.0 (0.0)	0.3 (0.3)	0.0 (0.0)	1.3 (1.7)
Jialeshuei	26.7 (10.4)	43.1 (12.2)	12.0 (3.4)	4.8 (2.6)	10.4 (3.4)	0.5 (0.9)	0.0 (0.0)	0.8 (1.9)	0.0 (0.0)	0.0 (0.0)	1 (0.7)	0.0 (0.0)	0.7 (0.5)
Longkeng	33.1 (4.5)	29.1 (1.6)	16.3 (4.2)	8.0 (4.5)	9.8 (2.2)	1.0 (0.4)	1.8 (1.7)	0.0 (0.0)	0.0 (0.0)	0.0 (0.0)	0.4 (0.4)	0.0 (0.0)	0.4 (0.3)
Leidashih	25.7 (7.5)	28.8 (12.1)	3.5 (3.8)	38.9 (20.2)	2.5 (2.5)	0.0 (0.0)	0.0 (0.0)	0.0 (0.0)	0.0 (0.0)	0.1 (0.1)	0.3 (0.3)	0.0 (0.1)	0.3 (0.4)
Sangjiaowan	52.7 (6.3)	24.6 (12.7)	14.9 (8.7)	2.0 (0.8)	3.6 (1.6)	0.1 (0.2)	0.5 (0.7)	0.0 (0.0)	0.0 (0.0)	0.0 (0.0)	1.1 (0.5)	0.0 (0.0)	0.5 (0.4)
Tanzihwan	52.6 (7.2)	15.1 (4.0)	26.4 (4.6)	0.2 (0.2)	4.2 (1.8)	0.0 (0.0)	0.0 (0.0)	0.0 (0.0)	0.0 (0.0)	0.0 (0.0)	0.6 (0.3)	0.0 (0.0)	0.9 (0.5)
Tiaoshih	80.2 (11.3)	11.8 (7.9)	4.3 (4.0)	0.7 (0.5)	1.5 (0.6)	0.0 (0.0)	0.1 (0.2)	0.0 (0.0)	0.0 (0.0)	0.0 (0.0)	1 (0.4)	0.0 (0.0)	0.4 (0.4)
Dingbaisha	78.3 (9.7)	4.8 (3.5)	10.2 (3.4)	0.2 (0.1)	0.1 (0.2)	0.0 (0.1)	0.2 (0.2)	0.0 (0.0)	0.2 (0.3)	0.0 (0.0)	0.3 (0.2)	0.0 (0.0)	5.7 (8.5)
Wanlitung	78.7 (3.6)	3.8 (1.0)	11.5 (2.8)	1.2 (1.4)	3.5 (1.1)	0.0 (0.0)	0.1 (0.1)	0.0 (0.0)	0.2 (0.2)	0.0 (0.0)	0.4 (0.3)	0.0 (0.0)	0.7 (0.5)
Hongchai	89.5 (1.4)	3.0 (1.0)	6.4 (0.7)	0.0 (0.0)	0.5 (0.5)	0.0 (0.0)	0.1 (0.1)	0.0 (0.0)	0.0 (0.1)	0.0 (0.0)	0.1 (0.2)	0.0 (0.0)	0.4 (0.2)
**TAIWAN [282 OTUs]**	**49.0 (26.0)**	**25.0 (17.7)**	**10.1 (9.8)**	**7.5 (15)**	**3.8 (4.6)**	**1.2 (5.0)**	**0.5 (1.0)**	**0.3 (1.5)**	**0.0 (0.1)**	**0.0 (0.0)**	**0.3 (0.4)**	**0.0 (0.1)**	**2.4 (4.8)**

*TU: Turf—F_(3,100)_ = 82.34, p < 0.001 / **HC**: Hard Coral—F_(3,100)_ = 31.75, p < 0.001 / **MA**: Macroalgae—F_(3,100)_ = 121.34, p < 0.001 / **SF**: Soft Coral—F_(3,100)_ = 84.53, p < 0.001 /**ECA**: Crustose Coralline Algae—F_(3,100)_ = 29.48, p < 0.001 / **ZO**: Zoanthid—F_(3,100)_ = 16.42, p < 0.001 / **SP**: Sponge—F_(3,100)_ = 2.12, p > 0.05 / **AN**: Anemone—F_(3,100)_ = 9.67, p < 0.001 / **AS**: Ascidian—F_(3,100)_ = 3.26, p < 0.05 / **AT**: Anthipatharia—F_(3,100)_ = 0.42, p > 0.05 / **OL**: Other Life—F_(3,100)_ = 20.16, p < 0.001 / **UN**: Unknown—F_(3,100)_ = 2.15, p > 0.05 / **BS**: Bare Substrate—F_(3,100)_ = 14.99, p < 0.01

Euclidean distances were then calculated between each combination of two sites, and multivariate patterns in benthic assemblages were visualized using non-metric multidimensional scaling (nMDS). The regional centroids (referring to spatial medians) were overlaid on the graphics. OTUs contributing up to 70% of the differentiation between regions were identified using a similarity percentage (SIMPER) routine and added to plots. A homogeneity of dispersion (PERMDISP) test was carried out to identify significant differences in within-group multivariate dispersion among regions [58]. The data did not conform to an assumption of homogeneity before and after transformation; therefore, testing for differences among regions using nested permutational multivariate analysis of variance (nested ADONIS) and analysis of similarities (ANOSIM) was performed on raw data using a conservative α value of 0.01. Bonferroni corrected pairwise comparisons (adjusted by the number of comparisons made) were further used to interpret any significant *p*-values. Both tests were used together as ADONIS is usually considered more conservative and less sensitive to dispersion than ANOSIM, which however, can be useful for analyzing the magnitude of divergence among regions. *P*-values were systematically generated from 9,999 random permutations in each case. The effect of latitude was then assessed by analyzing regional differences. In addition, latitude was used as a continuous predictor to test its effect on the benthic assemblage using the nested ADONIS test. However, this factor confounds the effect of seawater temperatures, which around Taiwan are not necessarily correlated with latitude due to the effect of the KC flowing along the east coast. Therefore, regional differences were considered the most pertinent factor in our analyses. All data were analyzed in R (v2.15.2) using the Vegan package [59].

## Results

A total of 25 sites, 125 transects, and 2,625 pictures were analyzed in this study. Benthic assemblages from all sites were categorized into 282 OTUs. Among these, 177 hard corals OTUs were distinguished, plus 29 of algae, and 19 of soft corals. Average α-diversity values were 55.8 ± 11.0 in north Taiwan, 47.8 ± 15.5 at Penghu, 87.0 ± 14.8 at Green Island, and 73.4 ± 22.0 at Kenting. OTU richness for these four regions were 104, 132, 164, and 200, respectively, with corresponding hard coral OTU richness values of 64, 91, 115, and 140.

Turf algae dominated benthic assemblages around Taiwan with an average coverage of 49.0 ± 26.0%. However, there were significant differences among the regions (nested ANOVA, F_3,100_ = 82.34, *p* < 0.001; Table 1), with Penghu presenting the highest coverage of turf algae (69.8 ± 16.6%), followed by Kenting (51.5 ± 26.0%), north Taiwan (38.9 ± 14.8%), and Green Island (20.8 ± 14.1%). Hard corals contributed 25.0 ± 17.7% to the hard substrate coverage, with Green Island (39.6 ± 11.0%) presenting a significantly higher hard coral coverage (nested ANOVA, F_3,100_ = 31.75, *p* < 0.001;Table 1) than the other three regions: Kenting (24.1 ± 19.0%), Penghu (21.0 ± 18.4%), and north Taiwan (18.8 ± 9.7%). Macro-algae (10.1 ± 9.8%) were significantly more abundant along northern Taiwan (23.2 ± 11.4%) than at Kenting (10.6 ± 7.7%) and Green Island (6.7 ± 5.4%), and less abundant at Penghu (2.4 ± 2.7%). Soft corals (7.5 ± 5.4%) were important components of the coverage at Green Island (23.8 ± 23.6%) and Kenting (7.8 ± 13.4%). The contributions of encrusting coralline algae, zoanthids, sea anemones, other benthic forms, and bare substrates showed significant differences among regions, but in each case represented less than 10% of coverage (Table 1).

**Fig 3:**
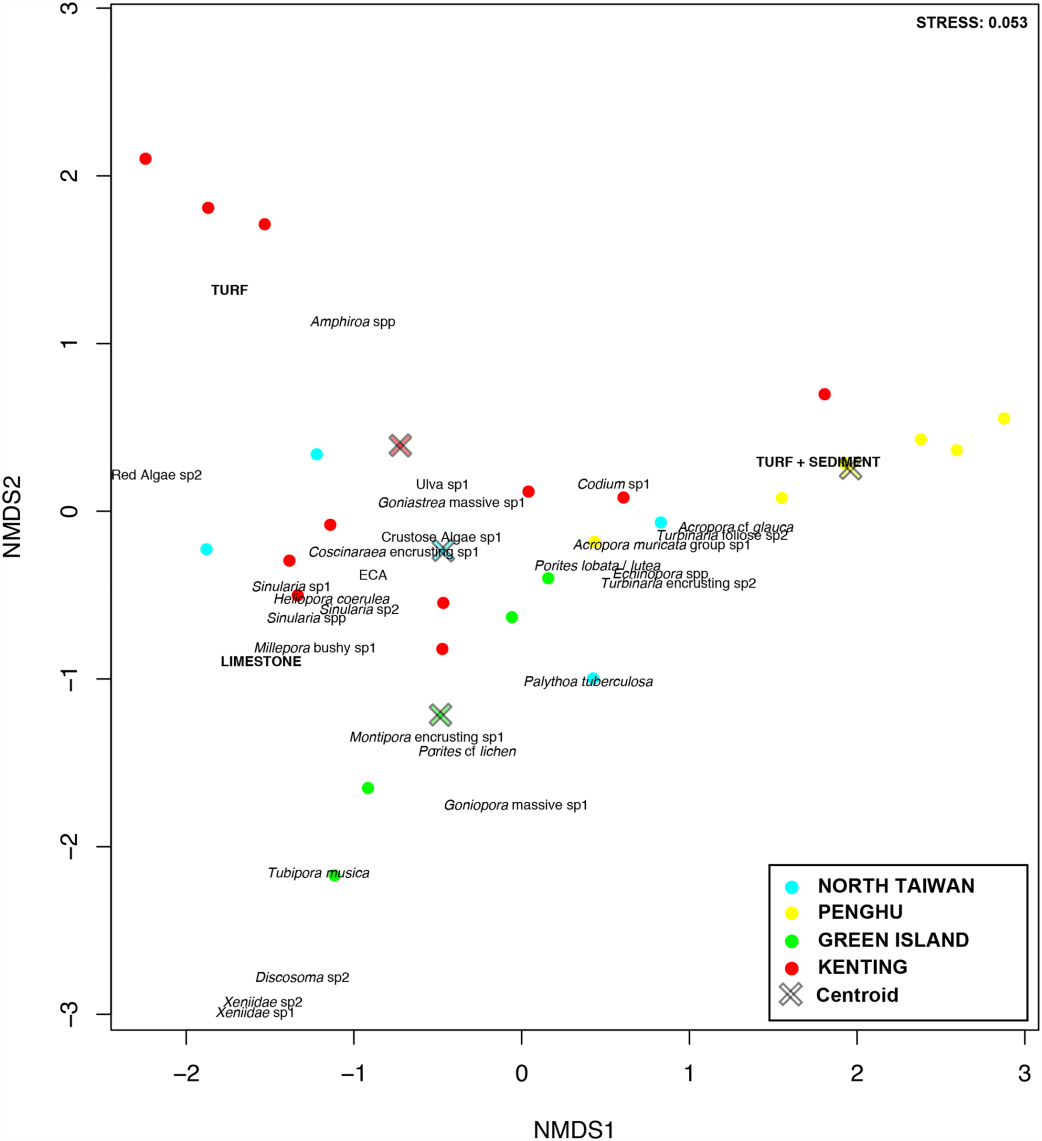
Multivariate pattern. nMDS ordination showing regional partitioning. Centroids (crosses) were overlaid on the multivariate pattern to represent the relationship between sites and regional dispersion, respectively. OTUs contributing up to 70% of the regional differentiations were added.

**Fig 4:**
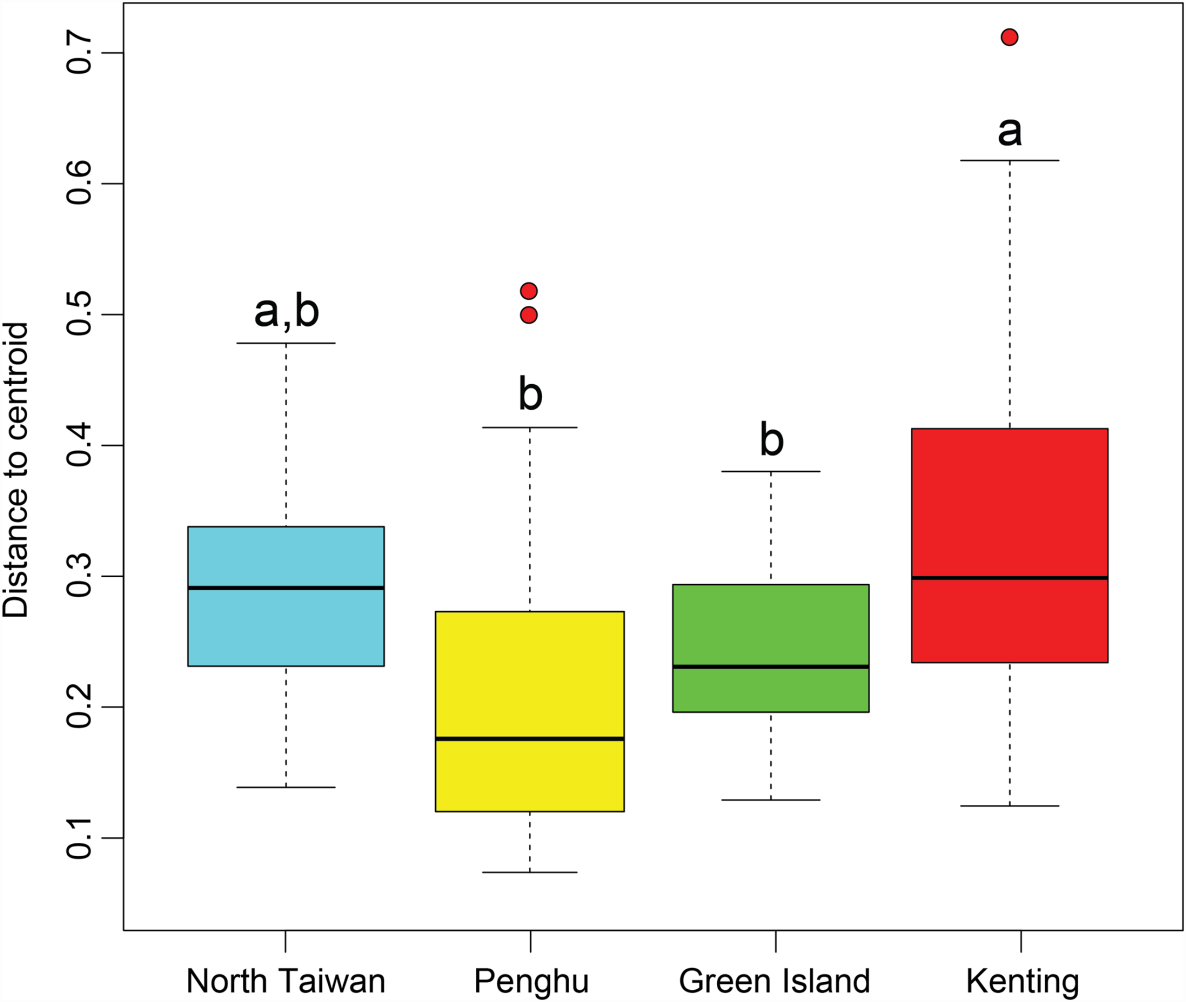
Boxplots summarizing, for each region, the distance of transects to regional centroids. Boxplots display median, first, and third quartiles (hinges); 95% confidence interval of median (notches) and outliers.

The benthic community structure within each region was clearly heterogeneous (Fig 3, S1 Fig). However, there were differences between regions (PERMDISP, F_3,121_ = 7.00, *p* < 0.001), with Kenting presenting significantly higher dispersion than Green Island and Penghu. North Taiwan was in an intermediate situation (Fig 4). Multivariate patterns showed that regional groups could be easily distinguished, but with obvious overlap among regions (Fig 3). The centroid of Penghu’s sites was distinctly separate from those of the other three regions. Based on shared occurrences of some hard and soft coral taxa, encrusting coralline algae, and some macro-algae, the differentiation followed three trajectories: (1) an occurrence of turf algae mainly represented by some transects in Kenting, (2) an occurrence of turf algae partially covered by sediment, especially at Penghu, and (3) an occurrence of Xeniidae corals, with *Tubipora musica* and the corallimorpharian *Rhodactis indosinensis* at Green Island. Two groups were easily distinguished at Kenting, one being dominated by turf algae and the other by hard corals (Table 1; Fig 3; S1 Fig). However Leidashih on the western side of Nanwan bay (Kenting) was pulled from this group, due to the high contribution of soft corals in this location, which usually characterizes transects from Green Island (Table 1). At Penghu, Wa En Tung was dominated by hard corals, strongly differing from all other sites in the region where sediment and turf algae typically represented > 60% of substrate coverage (Table 1, S1 Fig). The benthic assemblages of north Taiwan tended to include each of these features in part (Fig 3, S1 Fig). The regional centroid was positioned almost at the orthocenter of the triangle composed by the centroids of the other three regions. Longdon differed from other northern sites by having less macro-algae and encrusting coralline algae but more hard corals. Keelung Island was characterized by a higher contribution of plate-like corals, when Yeliu by the one of the zoanthid, *Palythoa tuberculosa*.

A significant difference in benthic assemblages was recorded with latitude (nested ADONIS, F_1,100_ = 38.94, *p* < 0.001), but this explained only 5% of observed variance. Regional difference (F_3,100_ = 100.73, *p* < 0.001, nested ADONIS) was better supported, with 40% of the variation in the benthic assemblage explained by this factor. Similarly, the ANOSIM test suggested a low but significant difference in the structure of benthic communities among regions (R = 0.34, *p* < 0.001), clearly indicating some overlap between groups. ANOSIM pairwise comparisons revealed that Penghu differed strongly from Green Island (R = 0.75, *p* < 0.001) and moderately from north Taiwan (R = 0.62, *p* < 0.001) and Kenting (R = 0.48, *p* < 0.001). Green Island differed weakly from north Taiwan (R = 0.27, *p* < 0.001) and Kenting (R = 0.16, *p* < 0.05). In contrast, benthic community structure was similar in the north and south (R = 0.05, *p* = 0.99).

## Discussion

This study was the first integrated research project conducted on benthic communities from multiple regions of Taiwan. Benthic assemblage structure showed only a weak differentiation between regions and along the latitudinal gradient previously identified in the diversity of some taxa. We hypothesize that local patterns of disturbance could be responsible for the heterogeneity observed at the regional scale, and may smooth out the differences between regions. We discuss below the potential role that this marine hotspot could play in the future, and highlight that the management of local factors responsible for reef degradation should remain a priority in order for marine ecosystems to cope with climate change.

### Structure of major benthic categories

Like general worldwide trend of reef degradation [60, 61], Taiwanese benthic communities were found to be largely dominated by turf algae (49.0 ± 26.0%). However, mean hard coral coverage was consistent with data previously reported from Taiwan and Japan (~24%, [62]), and slightly above the average coral coverage reported from the Indo-Pacific region in 1997–2004 (22.1%, [62]). Although comparison with those data should be interpreted with caution due to different protocols used and sites surveyed, it could indicate a stabilization to a low level of coral coverage in this area as this one was estimated to 50% in the past (i.e. 100–1000 yr BP) [62]. A similar trend was observed at the regional level, such as at Kenting, where Mok [63], suggested the stabilization (or even a slight increase) of coral coverage. Coral coverage was higher at Green Island and Kenting, consistent with the presence of well-developed fringing reefs [64] inhabited by a rich coral fauna (reviewed in [30]). Using coral coverage as an indicator of coral reef health [62], Green Island reefs could be relatively resilient to human disturbances, as characterized by an important influx of tourists during the summer (estimated at 400,000 visitors per year in 2002, [40]). In Kenting, anthropogenic disturbances were considered the major factors of degradation, producing heterogeneous ecological conditions in the reefs along the coastline [40, 44, 53]. Penghu showed the highest coverage of turf algae, which is coherent with the pattern of degradation observed from 1994 to 2004, at four reefs around the main island of the archipelago [65]. With a subtropical climate prevailing in northern Taiwan, the coverage of macro-algae (23.2 ± 11.4%) and encrusting coralline algae (8.3 ± 7.6%) were the highest compared to other surveyed regions (Table 1). This was coherent with the pattern of diversity around Taiwan [63, 66] and previous observations from high-latitude areas [67, 68].

### Diversity and multivariate patterns

An increasing diversity gradient was observed from North to South. Mean α-diversity and regional OTU richness values both suggested an obvious distinction in OTU richness between reefal (Kenting and Green Island) and non-reefal (Penghu and north Taiwan) benthic assemblages. As shown in [33], hard coral followed the same general pattern observed in diversity, with species richness increasing as SST rises and latitude decreases. In contrast, macro-algae was more diverse at the northeastern coast [31], consistent with its subtropical environment.

This latitudinal gradient in diversity became less evident when the ‘OTUs’ contribution to the overall community was taken into consideration. Regional groups clearly overlapped in the multivariate pattern, with highly heterogeneous benthic assemblages characterizing the different regions. A common pool of species across regions included various species of hard and soft coral taxa, encrusting coralline algae, and macro-algae. We hypothesize that this feature could be representative of reefs in Taiwan. A prevalence of functionally distinct organisms (see discussion in [69] and [70]) could also indicate resilient sites. Dispersion from this common facet followed three main trajectories: (1) a dominance of turf algae, particularly in Kenting, (2) a prevalence of turf covered by sediment, mostly characterizing Penghu, and (3) communities marked by the presence of soft corals (*Xeniidae* spp.), together with *Tubipora musica* and the corallimorpharian *Rhodactis indosinensis* at Green Island. In these different locations, regional characteristics (turf in Kenting, turf + sediment in Penghu and soft coral in Green Island) have been previously highlighted at some sites such as at Wanlitung in Kenting [42], Chinwan Inner Bay in Penghu [45], and the western and southwestern coasts of Green Island [71]. For example, the coverage of the zoanthid *Palythoa tuberculosa*, together with *Turbinaria* corals and macro-algae such as *Ulva* or *Sargassum*, were among the main contributors of the differentiation of the northern sites. *Halimeda*, *Amphiroa*, *Codium*, and *Neomeris* algae were typical of reef communities from Kenting and Green Island. Overall, macro-algae seemed to be better ecological indicators than corals in differentiating regions along a latitudinal gradient.

However,benthic assemblages were poorly differentiated by latitudinal (5% of the observed variance) or regional (up to 34–39% of the observed variance) factors. Overall, the obvious latitudinal gradient observed in species richness within taxa, including corals [33] and algae [31], was not well reflected in community structure. This was best illustrated by the assemblages in northern Taiwan and Kenting. They were the most similar despite being ~350 km apart and had contrasting seawater temperatures (annual average difference of 3.1°C). In addition, there was a weak but significant difference between the two closest locations, Kenting and Green Island. While shifts in benthic communities have been classically reported along a latitudinal cline [72–74], observations similar to those reported here were recorded along the Australian western coast, from 28.5°S to 33.5°S. There, it has been suggested that the attenuation of the latitudinal temperature gradient (2°C over 1,000 km of north-south coastline) by the Leeuwin Current (LC) enhances the influence of local factors in the patterns observed [68]. Therefore, Smale *et al*. [68] proposed that the selected locations, and possibly also the relatively coarse taxonomic resolution, could explain the lack of a latitudinal gradient in the benthic assemblages along the western coast of Australia. We hypothesize that the local anthropogenic and natural factors that are responsible for Taiwan’s regional heterogeneity, could have caused the situation observed in this study. Indeed, small scale variability triggered by local factors or disturbances may prevent us from distinguishing possible latitudinal patterns in benthic community structure. Further investigation at a regional scale is necessary to understand such local variability.

### Disturbance patterns

Taiwan is regularly affected by typhoons (averaging 4.5 typhoons per year [75]), and their frequency and intensity have tended to increase in recent decades [76]. According to historical paths, southern Taiwan has been the most susceptible part of the island to typhoons [77]. Aside from catastrophic damage to reefs by strong waves (see review in [78]), coral reef organisms suffer from terrestrial freshwater runoff following typhoons [78]. Reefs at Green Island seem to have recovered reasonably from past bleaching episodes, such as the 1998 event [79]. However, past severe mechanical disturbances generated by typhoons are suspected to have triggered a shift from hard- to soft coral-dominated communities on the west coast of Green Island [80], consistent with our observations at Shihland, and to a lesser extent at Dabaisha.

The effect of typhoons on benthic communities in Kenting seems not to be limited to mechanical damage, but to have a wide range of indirect impacts [78], such as increases in turbidity, sedimentation, and pollution, plus salinity decreases due to discharges from three major rivers [44]. On the west coast of the Hengchun Peninsula (Kenting), for example in Wanlitung, benthic communities suffer from recurrent and synergetic disturbances [42]. Typhoons, bleaching events, and locally-induced human stressors are responsible for the more than 50% decrease in coral coverage over the last 25 years and an increase by almost three fold in macro-algal coverage [42]. In Tiaoshih, those disturbances could also be responsible for the temporary shift from a coral- (*Acropora*) to the sea anemone- (*Condylactis*) dominated community that was observed in 2000–2003 [18]. The population of this anemone seems to have declined [81], as *Condylactis* was not observed during our survey despite the persistence of local anthropogenic disturbances. Coral coverage during our survey was 3.5- to 4.0-fold lower than in 1987 and 1997, respectively [44, 82, 83]. A mosaic of zones, with or without recovery trajectories toward the original *Acropora* community or other branching species such as *Montipora stellata*, were identified at this site and related to a variety of prevailing environmental stressors [81]. Human activities related to agriculture, land use, coastal development due to tourism, and overfishing have created diverse facets of reef degradation at Kenting [55], and could explain the lack of recovery at some sites. Liu *et al*. [44] showed that increases in the stability/quality of seawater is correlated to its distance from stream discharges. This significantly affects reef conditions at sites close to river mouths, which have lower percentages of coral coverage and higher one of macro-algae [44]. In Nanwan Bay, the coverage of macro-algae such as *Codium* is related to nutrient enrichment [30, 84, 85]. In our survey, the highest coverage of this macro-algae was observed in Sangjiaowan (6.7%) and Tanzihwan (5.5%), in the vicinity of the main river flowing into the bay (see S2 File). Together with Tiaoshih, these sites presented the highest percentages of turf and macro-algae in the Bay. While on the west coast of Nanwan, sheltered from direct freshwater discharges, Leidashih, Outlet, and Houbihu are characterized by more stable water quality [44]. There, coral (HC and SF together) and algae (TU and MA together) coverages were respectively the highest and the lowest in Kenting. These sites could also have benefitted from coastal development being restricted due the presence of a nuclear power plant, operational since 1985. The occurrence of tidal upwelling [86], unevenly affecting Nanwan [87], could strongly influence the response of reef organisms [88] and be responsible for the heterogeneity observed in the benthic assemblages [55]. Sites on the west coast of Kenting (Wanlitung, Hongchai, and Dingbaisha) do not seem to respond to the pattern of degradation associated with river discharge. The lowest coral coverage for Taiwan, as well as the prevalence of algae observed at these sites, could be due to alternative stressors such as those mentioned earlier for Wanlitung [42]. Overall, the effectiveness of the marine protected area, since its implementation in 1984 around the Hengchun peninsula, is relative, as illustrated by the contrasting situations observed in the no entry and no-take areas of KNP such as Sangjiaowan or Houbihu and Tiaoshih (S1 Table) [42, 55].

At Penghu, winter surges of cold water can cause mass mortality of reef organisms, as occurred during the 2008 cold shock event [49]. The high proportion of recently dead corals covered by turf algae at Cimei, Pon-Pon-Tan, and Siyuping (see Table 1) suggest that a similar event could have occurred again in Penghu during the winter of 2011–2012, when SST fell below 18°C for 40 consecutive days (HJ Hsieh, unpublished data). The high density of coral juveniles (< 5 cm, data not shown) at some locations, however, suggested a potential for fast recovery, which could be limited at some sites by human activities. For example, in Chinwan Inner Bay, coral coverage has suffered nutrient enrichment, sedimentation, overfishing [48], algae overgrowth [45], and an outbreak of the corallivorous snail *Drupella* [89]. In 2001, its coral coverage ranged between 63.9 and 93.0% [90], dramatically decreasing to 16.3% in 2008 [89] despite the establishment of a no-take area in the bay in 2005. In the present study, corals contributed only 5.4% of substrate coverage.

High-latitude sites seem to develop into monospecific community (see example in [91, 92]). Aside from natural and human stressors, we suggest that dispersion among sites in northern Taiwan could be a response to environmental conditions in this area. In the absence of external disturbances to coral assemblages, the α-diversity ratio could be decreased by an increase in latitude and a decrease in SST. Alternatively, the heterogeneity in this region may also be related to its location within a transition area between tropical and temperate locations. This was made particularly obvious by the co-occurrence of tropical and temperate organisms (especially for algae such as *Padina*, *Sargassum*, and *Ulva* species).

### The role of Taiwanese benthic communities under climate change conditions

Although the possibility of reefs surviving climate change via poleward migration is still debated [21, 93] and could be severely constrained by photosynthetically available radiation in winter [94], there is increasing evidence of the expansion of several tropical organisms towards higher latitudes [24, 92, 95]. In this scenario, the unique biogeographical overlap of temperate and tropical marine taxa in Taiwan could be threatened by rising seawater temperatures. In our study, the lack of an obvious latitudinal cline in benthic assemblages could be a result of the tropicalization and homogenization of marine communities around the island, smoothing out regional differences, but this process could be accompanied by a loss of species less tolerant to high temperatures. Similar phenomena have been recorded in the North Sea for six fish species (*Trisopterus luscus* and *esmarkii*, *Micromesistius poutassou*, *Echiichthys vipera*, *Glyptocephalus cynogloscus*, and *Arnoglossus lanterna*) [96]. Today, environmental constraints limit reef formation to the eastern and southern coasts of Taiwan, but in the mid-Holocene (6,000–5,000 years ago), extensive reefs occurred at Keelung (north Taiwan) when seawater temperatures were less than 2°C higher than at present [97, 98]. At Keelung Island, the contribution of pioneer and opportunistic frame-building species such as *Acropora hyacinthus* [92, 99] suggest that a benthic community providing a basis for a reef could eventually develop.

Nevertheless, there is no evidence to date suggesting that any taxa, including corals, have disappeared or moved poleward to benefit from recent changes in environmental conditions in Taiwan. However, Taiwan constitutes an important area for the connectivity of marine organisms in the Kuroshio region, and the degradation of benthic communities could therefore represent an important limitation to a possible poleward migration. If we establish high-latitude refuges for the survival of tropical taxa, propagule source conservation should remain a priority by reducing additional stressors in existing coral communities.

Overall, this is the first study suggesting that there is a generalized degradation of benthic assemblages around Taiwan. While recurrent natural disturbances probably explain part of the variation observed, anthropogenic disturbances are likely playing major roles at the local scale in explaining the heterogeneity observed within the region. Given the key geographic location of Taiwan in the Kuroshio region, this study highlights the importance of reinforcing marine conservation around the island and maintaining resilient coral assemblages to face climate change. This study provides the first integrative picture of the status of the benthic assemblages of the four main coral regions around Taiwan, and therefore constitutes an important baseline upon which future changes can be compared. Therefore, data and R script are provided as supplementary data for additional studies.

### Ethics statement

Permits numbered 10200401200 from Kenting National Park, 1024150391 and 10300200398 from Keelung city government, and 1034150073 and 1034100291 from the Fishing Management Office of New Taipei city government were required to perform field surveys in some restricted areas. All surveys procedures were non-destructive and were done with appropriate precautions to prevent damage to the ecosystem.

## Supporting Information

S1 Fig Taiwanese benthic assemblages—site location and protection status. Sites information (GPS coordinates in decimal degrees).
(TIF)

S1 File Taiwanese benthic assemblages—script for data analysis. (TXT)

S2 File Taiwanese benthic assemblages—dataset. (CSV)

S3 File Taiwanese benthic assemblages—dataset for nested ANOVA on Major Categories. (CSV)

S4 File Taiwanese benthic assemblages—regional and site factors. (CSV)

S5 File Taiwanese benthic assemblages—classification of OTUs into major categories. (CSV)

S1 Table Taiwanese benthic assemblages—Multivariate pattern. nMDS ordination showing regional partitioning at transect level. Centroids (crosses) were overlaid on the multivariate pattern to represent the relationship between transects and regional dispersion, respectively. OTUs contributing up to 70% of the regional differentiations were added.
(DOCX)
