# Supplementary figures and images for: Structure of Benthic Communities along the Taiwan Latitudinal Gradient
Source: PLoS One. 2016 Aug 11;11(8):e0160601. doi: 10.1371/journal.pone.0160601 (PMC4981444; doi:10.1371/journal.pone.0160601)

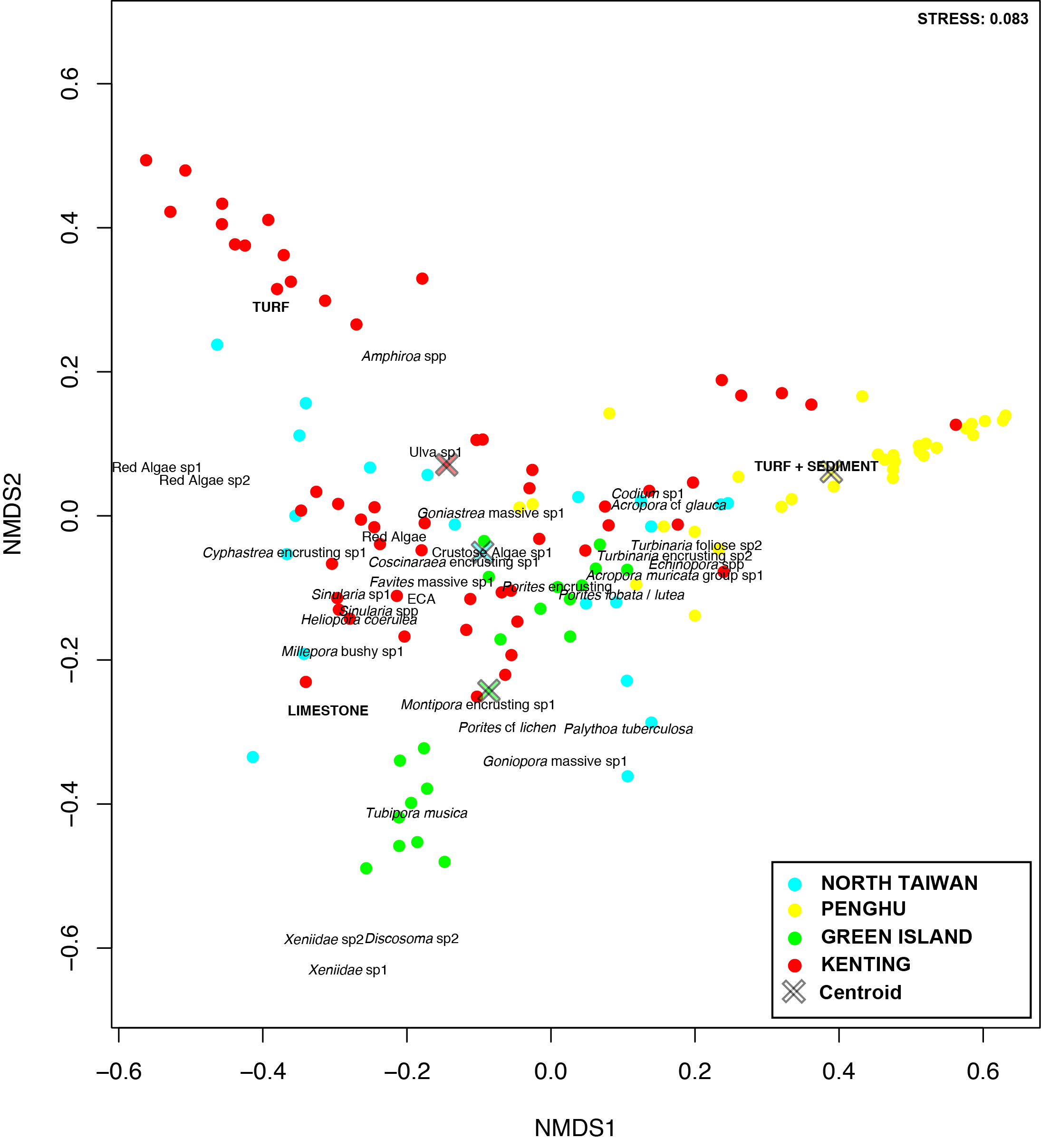

Supplement: S1 Fig — Sites information (GPS coordinates in decimal degrees). (TIF) [file pone.0160601.s001.tif]
