# Supplementary material for: Structure of Benthic Communities along the Taiwan Latitudinal Gradient
Source: PLoS One. 2016 Aug 11;11(8):e0160601. doi: 10.1371/journal.pone.0160601 (PMC4981444; doi:10.1371/journal.pone.0160601)
Supplement: S1 Table — nMDS ordination showing regional partitioning at transect level. Centroids (crosses) were overlaid on the multivariate pattern to represent the relationship between transects and regional dispersion, respectively. OTUs contributing up to 70% of the regional differentiations were added. (DOCX) [file pone.0160601.s007.docx]

| Site | Region | Latitude | Longitude | National Parks | National Scenic Areas | Geological Parks | Taiwan No take and No Entry Areas |
| --- | --- | --- | --- | --- | --- | --- | --- |
| Keelung Island | North Taiwan | 25.19107 | 121.7851 | - | **X** | - | - |
| Yeliu | North Taiwan | 25.20767 | 121.69437 | - | **X** | **X** | - |
| Bitou | North Taiwan | 25.12577 | 121.9156 | - | **X** | **X** | No Take |
| Longdon | North Taiwan | 25.11327 | 121.9202 | - | **X** | - | - |
| Wa En Tung | Penghu Archipelago | 23.537045 | 119.614497 | - | **X** | - | - |
| Gupoyu | Penghu Archipelago | 23.714767 | 119.5521 | - | **X** | - | - |
| Siyuping | Penghu Archipelago | 23.27525 | 119.5028 | **X** | **X** | - | - |
| Pon Pon Tan | Penghu Archipelago | 23.6725 | 119.638733 | - | **X** | - | - |
| Cimei | Penghu Archipelago | 23.188898 | 119.425418 | **X** | **X** | - | - |
| Chinwan Inner Bay | Penghu Archipelago | 23.533342 | 119.567151 | - | **X** | - | No Take |
| Gongguan | Green Island | 22.67762 | 121.49258 | - | **X** | - | - |
| Chaikou | Green Island | 22.67695 | 121.47868 | - | **X** | - | - |
| Dabaisha | Green Island | 22.63871 | 121.492158 | - | **X** | - | - |
| Shihland | Green Island | 22.654746 | 121.473702 | - | **X** | - | - |
| Houbihu | Kenting | 21.94305 | 120.75198 | **X** | - | - | No Take |
| Outlet | Kenting | 21.931367 | 120.74463 | **X** | - | - | - |
| Jialeshuei | Kenting | 21.99923 | 120.87312 | **X** | - | - | - |
| Longkeng | Kenting | 21.90775 | 120.86127 | **X** | - | - | No Entry |
| Leidashih | Kenting | 21.930117 | 120.74482 | **X** | - | - | - |
| Sangjiaowan | Kenting | 21.92355 | 120.82933 | **X** | - | - | No Entry |
| Tanzihwan | Kenting | 21.95 | 120.775 | **X** | - | - | No Take |
| Taioshih | Kenting | 21.958 | 120.766 | **X** | - | - | No Take |
| Dingbaisha | Kenting | 21.942417 | 120.70973 | **X** | - | - | No Entry |
| Wanlitung | Kenting | 21.995017 | 120.7036 | **X** | - | - | No Take |
| Hongchai | Kenting | 21.972533 | 120.71517 | **X** | - | - | - |
